# Improve Cardiac Emergency Preparedness by Building a Team-Based Cardiopulmonary Resuscitation Educational Plan

**DOI:** 10.3389/fpubh.2022.895367

**Published:** 2022-07-07

**Authors:** Jianing Xu, Xuejie Dong, Hongfan Yin, Zhouyu Guan, Zhenghao Li, Fangge Qu, Tian Chen, Caifeng Wang, Qiong Fang, Lin Zhang

**Affiliations:** ^1^School of Public Health, Shanghai Jiao Tong University, Shanghai, China; ^2^School of Nursing, Shanghai Jiao Tong University, Shanghai, China; ^3^Department of Global Health, School of Public Health, Peking University, Beijing, China

**Keywords:** out-of-hospital cardiac arrest, cardiopulmonary resuscitation, bystander, teamwork, emergency preparedness

## Abstract

**Objective:**

To design an innovative team-based cardiopulmonary resuscitation (CPR) educational plan for multiple bystanders and evaluate whether it was associated with better teamwork and higher quality of resuscitation.

**Methods:**

The team-based CPR plan defined the process for a three-person team, emphasize task allocation, leadership, and closed-loop communication. Participants qualified for single-rescuer CPR skills were randomized into teams of 3. The teamwork performance and CPR operation skills were evaluated in one simulated cardiac arrest scenario before and after training on the team-based CPR plan. The primary outcomes were measured by the Team Emergency Assessment Measure (TEAM) scale and chest compression fraction (CCF).

**Results:**

Forty-three teams were included in the analysis. The team-based CPR plan significantly improved the team performance (global rating 6.7 ± 1.3 vs. 9.0 ± 0.7, corrected *p* < 0.001 after Bonferroni's correction). After implementing the team-based CPR plan, CCF increased [median 59 (IQR 48–69) vs. 64 (IQR 57–71%)%, corrected *p* = 0.002], while hands-off time decreased [median 233.2 (IQR 181.0–264.0) vs. 207 (IQR 174–222.9) s, corrected *p* = 0.02]. We found the average compression depth was significantly improved through the team-based CPR training [median 5.1 (IQR 4.7–5.6) vs. 5.3 (IQR 4.9–5.5) cm, *p* = 0.03] but no more significantly after applying the Bonferroni's correction (corrected *p* = 0.35). The compression depths were significantly improved by collaborating and exchanging the role of compression among the participants after the 6th min.

**Conclusion:**

The team-based CPR plan is feasible for improving bystanders teamwork performance and effective for improving resuscitation quality in prearrival care. We suggest a wide application of the team-based CPR plan in the educational program for better resuscitation performance in real rescue events.

## Introduction

Health literacy is widely regarded as essential for achieving health and well-being (1, 2). As an essential part of health literacy, governments worldwide have adopted national strategies and targets to improve emergency preparedness (3, 4). It is noted that out-of-hospital cardiac arrest (OHCA) is one of the most critical medical emergencies. Prompt and effective cardiopulmonary resuscitation (CPR) by community first responders is essential for a better prognosis for patients with OHCA (5). To achieve high-quality CPR, China has proposed to have 3% and upper of its personnel certified in CPR training by 2030.

On account of complex procedures in resuscitation, the engagement of multiple rescuers is indispensable to guarantee high-quality CPR for patients with OHCA. It has been revealed that the presence of multiple bystanders is an independent factor positively correlated with high-performance CPR (6). Notably, 43.1% of OHCA rescue events are with multiple bystanders on the scene, which enables the implementation of the resuscitation procedures by teams of responders instead of isolated rescuers (7). Under these circumstances, a better prognosis for patients with OHCA is not only achieved by the high-quality CPR skills of every single rescuer but also by the cooperation of multiple bystanders to provide early defibrillation and avoid interruptions in resuscitation (8, 9). What is more, in prolonged resuscitation which is time-consuming and labor-intensive, team cooperation could enhance the quality of CPR while relieving the fatigue of individual rescuers (10).

Despite the high likelihood and feasibility of the resuscitation by multiple rescuers, it was rare to find standardized team educational plans and training programs for bystanders. Most of the established CPR educational plans for bystanders mainly emphasizing single-rescuer skills but neglect training in cooperation with others to implement better prearrival care (11). In advanced cardiovascular life support (ACLS), team-based resuscitation has been emphasized to improve survival and neurologic outcomes among cardiac arrest patients in the updated 2020 American Heart Association (AHA) and 2021 European Resuscitation Council (ERC) guidelines (12, 13). There is a lack of consolidated educational plans to provide effective and efficient methods of cooperation and communication for bystanders in a resuscitation team. Hence, we aimed to design an innovative educational plan for team-based CPR to influence the CPR operation skills and the cooperation among multiple bystanders. Furthermore, we hoped it could be widely applied in the educational program for better emergency preparedness about OHCA in real rescue events.

## Materials and Methods

### Study Design and Ethics

We innovatively designed a team-based CPR educational plan for bystanders according to the updated AHA and ERC guidelines. Implemented quantitative analysis to evaluate the effectiveness and feasibility of the team-based CPR plan in a before-after intervention simulation study. Participants were randomly allocated to teams of 3 people to perform resuscitation in a controlled simulated scenario and then tested in the same scenario after receiving the training of our designed team-based CPR educational plan. Ethics approval has been obtained from the Joint Research Ethics Board of the Shanghai Jiao Tong University Schools of Public Health and Nursing (SJUPN-201913).

### Study Participants and Randomization

From 1 January 2020 to 15 June 2021, 135 medical school students over 18 years old were recruited for the current study. Participants were chosen from among students who had not participated in the clinical practice or CPR training experiment. First, individuals were taken the basic life support training hosted by WeCan CPR training program (14). Through the WeCan CPR training, participants mastered skills including calling the emergency dispatch number of China's mainland (1-2-0), performing chest compression, mouth-to-mouth ventilation, and using an automated external defibrillator (AED).

Then the team-based CPR training was conducted for the participants, who worked in groups of three allocated by a simple random sampling method. All participants were informed about the intention of the present study and provided written informed consent to participate in the study.

### Team-Based CPR Educational Plan

**Figure 1:**
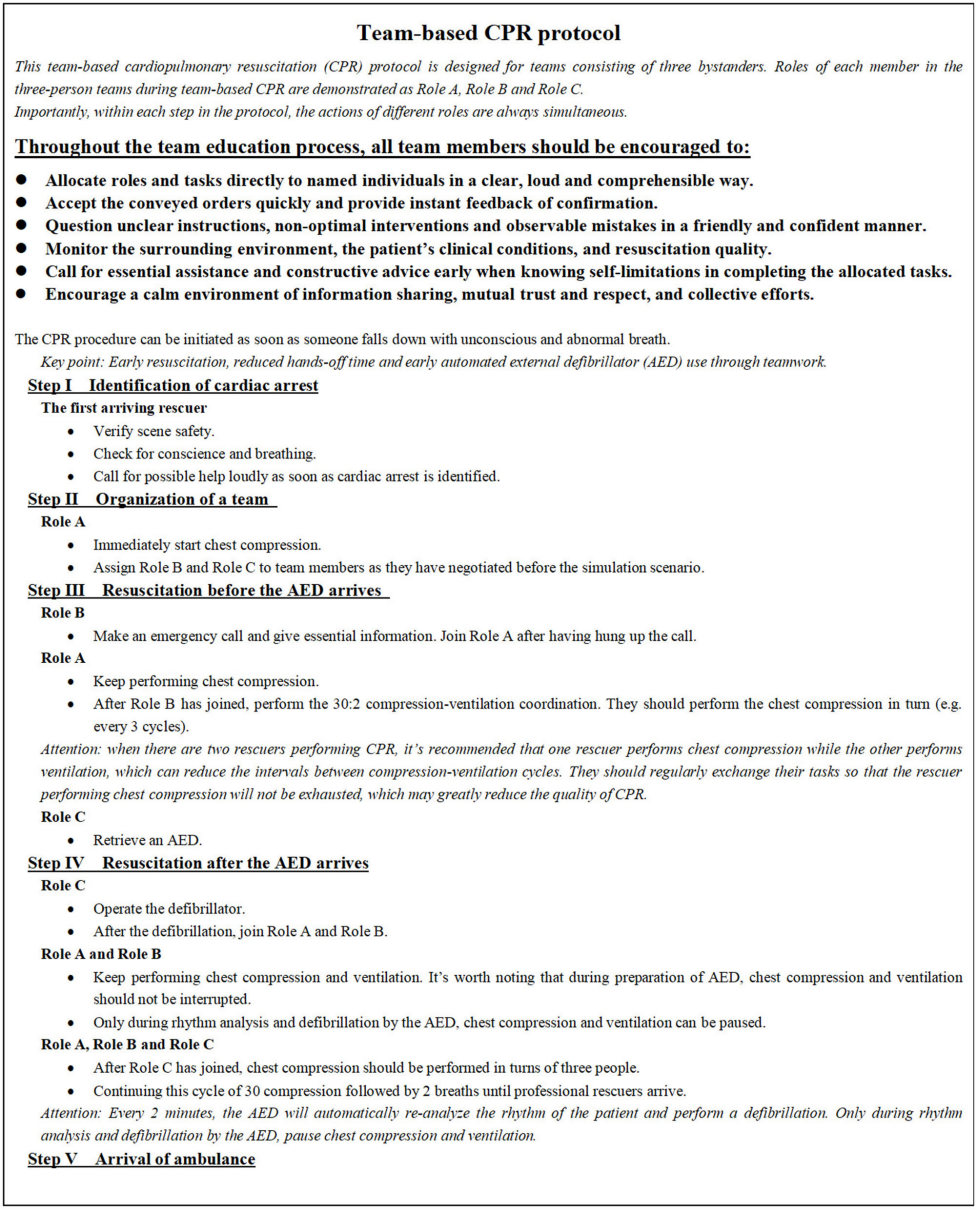
Team-based cardiopulmonary resuscitation protocol. CPR, cardiopulmonary resuscitation; AED, automated external defibrillator.

The innovative team-based CPR educational protocol was designed for teams consisting of three bystanders. The protocol referred to the Team Strategies and Tools to Enhance Performance and Patient Safety (TeamSTEPPS) and ACLS guidelines. TeamSTEPPS is a teamwork and communication systems model that has the potential to increase the effectiveness of team members with regard to leadership, communication, situation monitoring, and mutual support (15). As shown in Figure 1, the team-based CPR protocol divided the team-based CPR process into five parts, namely, (1) identification of cardiac arrest, (2) organization of a team (role distribution), (3) resuscitation before the AED arrives (Role A and Role B), (4) resuscitation after the AED arrives (Role A, Role B, and Role C), and (5) arrival of the ambulance. It concisely defined the roles and responsibilities of each member and emphasized the methods and importance of reasonable task allocation, leadership, and closed-loop communication within the resuscitation team. The protocol still highlights the critical elements of communication skills (call-out and check-back), cooperation skills (mutual support and knowledge sharing), and leadership skills (maintenance of a global perspective and task management).

The team-based CPR educational training contained a 10-min video course and a 20-min exercise. The 10-min video course strictly referenced the team-based CPR protocol and included an example demonstrating each step of it.

### Effectiveness Assessment of the Team-Based CPR Educational Plan

We designed a before-after intervention simulation study for lay rescuers. Each group of three individuals was asked to operate the OHCA resuscitation twice before and after the team-based CPR educational plan was applied in the simulated scenario. We evaluated whether the training with the team-based CPR protocol was associated with improving resuscitation quality and better-organized teamwork.

The team-based CPR simulated scenario was set in a quiet and isolated room with a manikin (Resusci Anne QCPR, Laerdal Medical, Stavanger, Norway) laid on the floor. Each team was required to assign and finish tasks including calling the emergency dispatcher, chest compressions, mouth-to-mouth ventilation, and retrieving and using an AED. The scenario started when each team walked into the room and stopped 10-min after they started the first chest compression. When Role C left and fetched the AED (Laerdal AED trainer 2, Laerdal Medical, Stavanger, Norway), he/she was required to return to the room after 5 min.

Before the simulated scenario started, researchers explained to the participants the tasks they needed to complete. No feedback was provided to the participants once the scenario started. The performance of each team throughout the entire scenario was recorded by three cameras from different angles: the head, the side, and the above.

### Data Collection and Outcome Measures

Data were collected from manikin feedback, video recordings, and the evaluation of researchers to measure the resuscitation quality and teamwork of each team. Outcomes were collected before and after the training and submitted to a blind assessor.

The primary outcomes were the Team Emergency Assessment Measure (TEAM) scale and chest compression fraction (CCF) (16). The TEAM scale consists of 11 items measuring the teamwork behaviors of medical teams dealing with critical situations. It has been used in several clinical and simulation-based studies with comparable outcomes and has been proven to be the most appropriate and valid tool for evaluating teamwork in emergency teams (17, 18). The TEAM scale involves three categories: leadership (two items), teamwork (7 items), and task management (two items). Each item is rated on a five-point Likert scale. And then, a final global rating scale of 1 to 10 was used as an overall comprehensive review of performance (19). Researchers were previously instructed about how to use the scale and provided with specific training on the scoring criteria. Two researchers evaluated the TEAM scale by watching video recordings of each team independently and discussed it to reach a consensus on the evaluation. CCF was calculated using the equation: 1—(real hands-off time/total scenario time), in which the hands-off time, including the delay at the beginning and all pauses, was measured in seconds using SimPad PLUS (Laerdal Medical, Stavanger, Norway).

Secondary outcomes included the quality of chest compressions, ventilation, AED performances, and time intervals during the resuscitation. The quality of chest compressions and ventilations were recorded using SimPad PLUS. AED performances were assessed by researchers using a dichotomous (yes/no) format. Time intervals during the resuscitation were measured using a stopwatch from the beginning of the simulated scenario. Participants' age, sex, and self-reported body weight were also documented.

### Sample Size and Power Analysis

The study sample size was calculated based on a pilot study of six teams, in addition to participant availability considerations. A 5% change in CCF was considered a relevant difference. With statistical power of 90% and a two-sided alpha level of 0.05, the minimum number of teams was found to be 35. We recruited 45 teams (135 participants), considering the possibility of loss to the post-training and the participants' availability. Those six teams in the pilot period were included in the final analysis as the data were homogeneity.

### Statistical Analysis

Data were presented as frequencies with percentages for categorical variables and means with SDs or median (interquartile range) for continuous variables. Differences in categorical outcomes were assessed using McNemar's test. The Normal distribution was confirmed using the Kolmogorov–Smirnov test. Differences between pre-training and post-training were compared using the Wilcoxon matched-pairs signed-rank test for continuous variables with non-parametric distribution and the paired sample *t*-test for continuous variables with normal distribution. We use the Bonferroni correction to control the type I error rate when multiple testing is performed (20). All statistics were managed by Office Excel 2010 and analyzed using SPSS 24.0. Two-sided *p*-values < 0.05 were considered significant.

## Results

**Figure 2:**
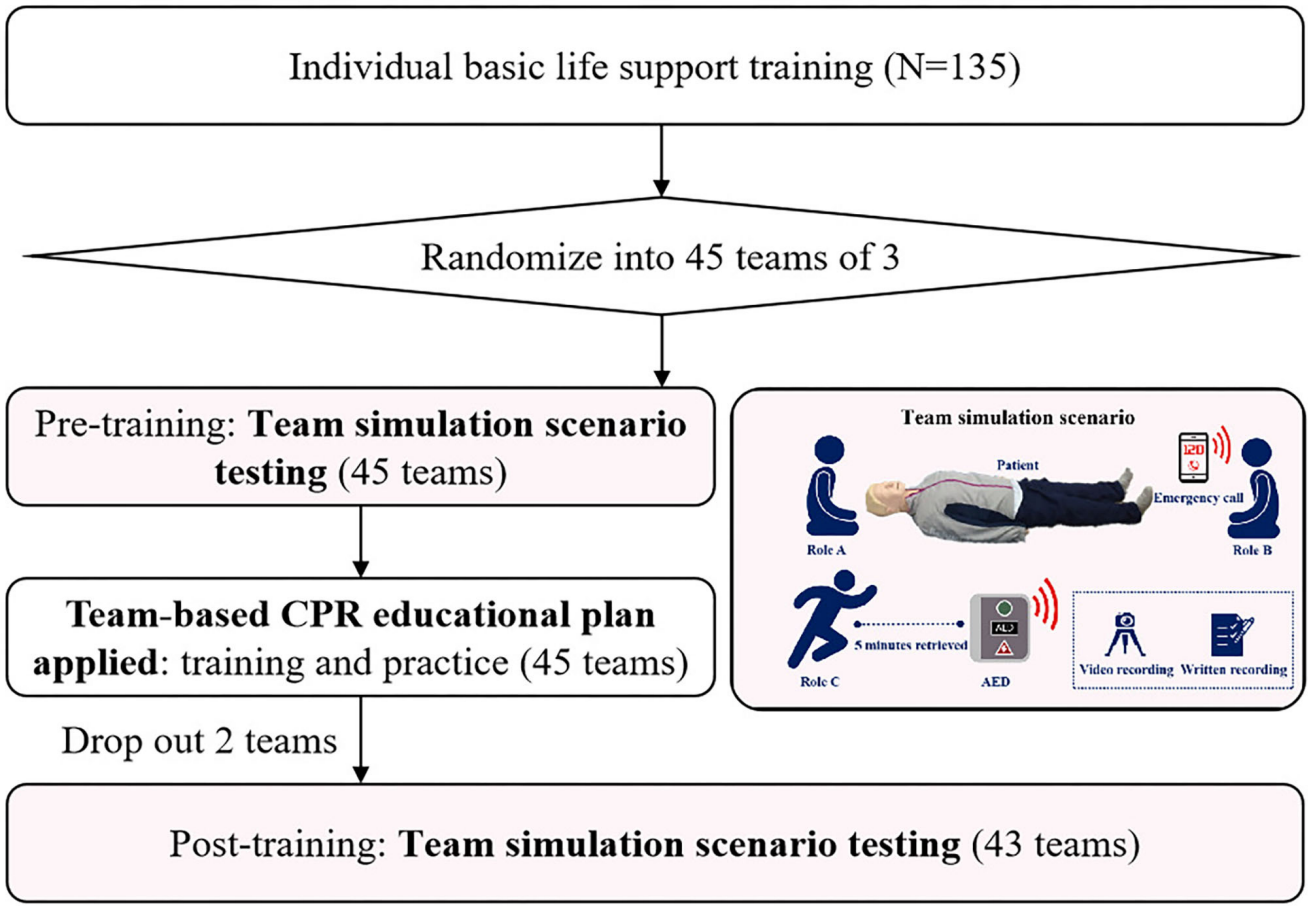
Flow diagram of the study. CPR, cardiopulmonary resuscitation.

We recruited 135 medical students and randomly divided them into 45 resuscitation teams. Two teams were excluded from analyses due to loss after pre-training simulation assessment for urgent business (Figure 2). A total of 43 three bystanders' teams consisting of 129 participants were included for analysis with a mean age of 20.86 years (SD = 2.54) and a mean weight of 58.39 kg (SD = 12.22). In total, twenty-eight (21.7%) of them were men. The demographic characteristics of participants such as age, weight, and gender, did not differ between those who completed the post-training and those who did not.

After the plan was applied, the global rating of teamwork performance was significantly improved (6.7 ± 1.3 vs. 9.0 ± 0.7, *p* < 0.001) with all team performance dimensions significantly improved (*p* < 0.05) (Table 1). After applying Bonferroni's correction, the global rating of teamwork performance was still significantly improved (corrected *p* < 0.001). The team-based CPR plan still significantly improved participants' abilities in all the leadership dimensions (2/2) and task management dimensions (2/2), and parts of the teamwork dimensions (5/7) by adjusting Bonferroni's correction (corrected *p* < 0.05).

**Table 1:** Comparison of the team-based CPR performance using Team Emergency Assessment Measure scale.

**Team performance**	**Pre-training** **(*n* = 43)**	**Post-training** **(*n* = 43)**	***P*-value^c^**	***P*_corrected_ ^d^**
**Leadership^a^**				
1. The team leader let the team know what was expected of them through direction and command	2.2 ± 1.5	3.6 ± 1.0	<0.001	<0.001
2. The team leader maintained a global perspective	2.4 ± 1.3	3.3 ± 1.0	<0.001	0.001
**Teamwork^a^**				
3. The team communicated effectively	2.9 ± 1.1	3.8 ± 0.4	<0.001	<0.001
4. The team worked together to complete tasks in a timely manner	2.6 ± 1.1	3.7 ± 0.6	<0.001	<0.001
5. The team acted with composure and control	2.9 ± 0.9	3.5 ± 0.7	0.003	0.04
6. The team morale was positive	1.8 ± 0.9	2.7 ± 0.6	<0.001	<0.001
7. The team adapted to changing situations	3.0 ± 1.1	3.9 ± 0.4	<0.001	<0.001
8. The team monitored and reassessed the situation	3.3 ± 1.5	3.9 ± 0.6	0.01	0.15
9. The team anticipated potential actions	2.3 ± 1.0	2.7 ± 1.0	0.03	0.37
**Task management^a^**				
10. The team prioritized tasks	2.9 ± 1.0	3.8 ± 0.6	<0.001	<0.001
11. The team followed approved standards/guidelines	2.9 ± 0.9	3.7 ± 0.4	<0.001	<0.001
**Overall^b^**				
12. Global rating	6.7 ± 1.3	9.0 ± 0.7	<0.001	<0.001

*Values were shown as mean ± SD*.

a *Task management items are rated on a 5-point Likert scale*.

b *The overall performance is rated on a global rating scale of 1–10*.

c *p-Values were derived by paired sample t-test*.

d *Bonferroni's correction for multiple testing*.

**Figure 3:**
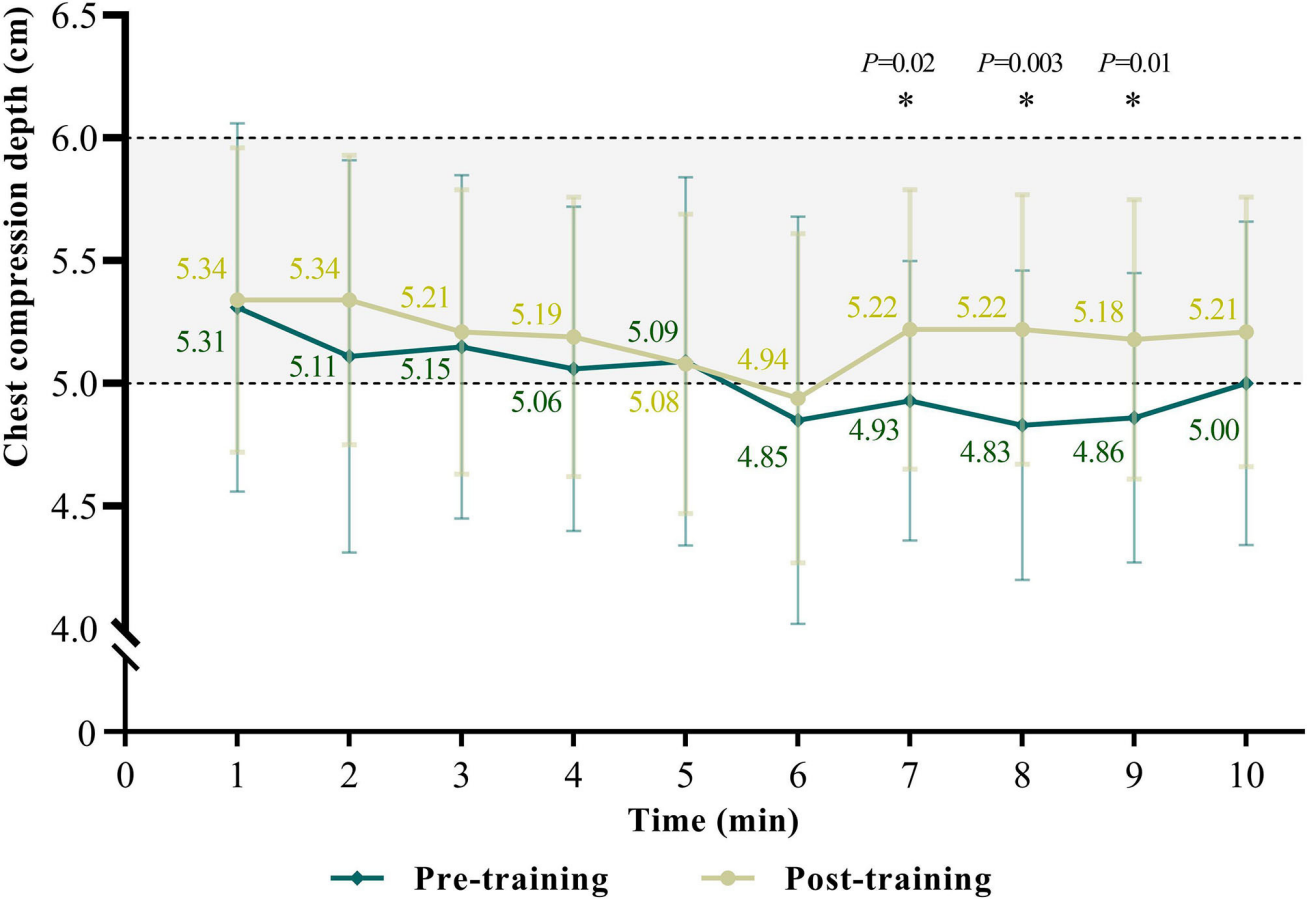
Trends of chest compression depths in team-based cardiopulmonary resuscitation scenario.

After the team-based CPR training, the CCF has significantly improved [median 59 (IQR 48–69) vs. 64 (IQR 57–71) %, *p* < 0.001] as hands-off time has decreased [median 233.2 (IQR 181.0–264.0) vs. 207 (IQR 174–222.9) s, *p* = 0.001] (Table 2). After applying Bonferroni's correction, the CCF and hands-off time remained significant (corrected *p* = 0.002 and corrected *p* = 0.02, respectively). The overall score for resuscitation performance was significantly improved [median 27 (IQR 18–47) vs. 49 (IQR 34–64)%, corrected *p* < 0.001]. We found the average compression depth was significantly improved through the team-based CPR training [median 5.1 (IQR 4.7–5.6) vs. 5.3 (IQR 4.9–5.5) cm, *p* = 0.03] but no more significantly by applying Bonferroni's correction (corrected *p* = 0.35). We found the compression depths were significantly improved by collaborating and exchanging the role of compression among the participants after the 6th min (Figure 3). The time points of compression depth were significantly improved at the 7th min (4.93 ± 0.57 vs. 5.22 ± 0.57 cm, *p* = 0.02), 8th min (4.83 ± 0.63 vs. 5.22 ± 0.55 cm, *p* = 0.003) and 9th min (4.86 ± 0.59 vs. 5.18 ± 0.57 cm, *p* = 0.01).

**Table 2:** Team-based CPR operation assessment by three bystanders.

**Parameters**	**Pre-training** **(*n* = 43)**	**Post-training** **(*n* = 43)**	***P*-value^b^**	***P*_corrected_ ^c^**
**Overall**				
CCF (%)	59.0 (48.0–69.0)	64.0 (57.0-71.0)	<0.001	0.002
Hands-off time (s)	233.2 (181.0–264.0)	207.0 (174.0-222.9)	0.001	0.02
Overall score (%, 100 in total)	27.0 (18.0–47.0)	49.0 (34.0-64.0)	<0.001	<0.001
**Chest compression**				
Average compression depth (cm)	5.1 (4.7–5.6)	5.3 (4.9–5.5)	0.03	0.35
Deep enough compression (5-6 cm) (%)	65.0 (36.0–82.0)	72.0 (50.0–86.0)	0.02	0.24
Average compression rate (min^−1^)	112.0 (107.0–118.0)	121.0 (116.0–124.0)	<0.001	0.001
Compression with adequate rate (100–120 min^−1^) (%)	75.0 (44.0–88.0)	51.0 (23.0–84.0)	0.02	0.24
Compression score (%, 100 in total)	76.0 (58.0–84.0)	82.0 (71.0–87.0)	0.005	0.06
**Ventilation**				
Average ventilation volume (ml)	518.0 (438.0–584.0)	570.0 (513.0–644.0)	0.02	0.26
Perform circles of 30 compressions and 2 breaths (%)^a^	31 (72.1)	38 (88.4)	<0.001	<0.001
Ventilation score (%, 100 in total)	55.0 (35.0–72.0)	65.0 (50.0–78.0)	0.06	0.79
**AED support**				
Placing the electrodes without flow time (%)^a^	31 (72.1)	41 (95.3)	<0.001	<0.001
Clear while AED delivers the shock (%)^a^	11 (25.6)	21 (48.8)	0.11	1.43

*Values were shown as median (interquartile range)*.

a *Values were shown as n (%). p-Values were derived by McNemar's tests*.

b *p-Values were derived by Wilcoxon matched-pairs signed rank test*.

c *Bonferroni's correction for multiple testing*.

*CPR, cardiopulmonary resuscitation; CCF, chest compression fraction; AED, automated external defibrillator*.

The emergency response time, including time to cardiac arrest identification [median 10 (IQR 7–16.3) vs. 7.0 (IQR 6–12) s, *p* = 0.001], time to open the airway [median 45.0 (IQR 36.0–63.0) vs. 39.0 (IQR 31–49) s, *p* = 0.01] and time to resume CPR aftershock [median 5.8 (IQR 3.9–8) vs. 3.5 (IQR 3–4.1) s, *p* < 0.001] were significantly shortened after the educational plan was applied (Table 3). After Bonferroni's correction, the indicators above remained significant (corrected *p* < 0.05).

**Table 3:** Team-based emergency response time assessment.

**Time intervals**	**Pre-training** **(*n* = 43)**	**Post-training** **(*n* = 43)**	***P*-value^b^**	***P*_corrected_ ^c^**
Time to cardiac arrest identification (s)	10.0 (7.0–16.3)	7.0 (6.0–12.0)	0.001	0.01
Time to call 1-2-0 (s)	14.0 (9.5–22.5)	12.0 (9.0–18.0)	0.05	0.28
Time to first chest compression (s)	18.0 (15.0–25.0)	15.0 (12.0–21.0)	0.001	0.01
Time to open the airway (s)	45.0 (36.0–63.0)	39.0 (31.0–49.0)	0.01	0.08
Time to first shock (s)^a^	381.0 (370.0–394.5)	378.0 (366.0–389.0)	0.05	0.32
Time to resume CPR after shock (s)	5.8 (3.9–8.0)	3.5 (3.0–4.1)	<0.001	<0.001

*Values were shown as median (interquartile range), p-Values were derived by Wilcoxon matched-pairs signed rank test*.

a *Including 5 min spent for the retrieval of AED*.

b *p-Values were derived by Wilcoxon matched-pairs signed rank test*.

c *Bonferroni's correction for multiple testing*.

*CPR, cardiopulmonary resuscitation; 1-2-0, emergency dispatch number of China's mainland; AED, automated external defibrillator*.

## Discussion

Optimal role assignment and effective cooperation in resuscitation are required when there are multiple bystanders on the scene of an OHCA. However, most of the established CPR training plans for bystanders only emphasize the technical skills of individual rescuers (21). As resuscitation effort is a time-consuming and labor-intensive task, it's indispensable to propose an educational plan to distribute the roles optimally and maximize the effectiveness of multiple bystanders, to guarantee the implementation of high-quality CPR for patients with OHCA. To our knowledge, this is the first study to innovatively design a team-based CPR educational plan for bystanders and implement quantitative analysis to evaluate its effectiveness and feasibility in a cardiac arrest simulated scenario. Our study demonstrated that team-based CPR training could significantly improve teamwork performance and resuscitation quality through positive and valid communication and cooperation. It increased the proportion of adequate compression depth, reduced hands-off time, avoided excessive ventilation, and ensured early defibrillation. The optimization of all these indicators would be theoretically associated with improved survival and neurological outcomes for patients with OHCA (7, 22, 23).

It was noted that, without team educational protocol, a group of three participants could not achieve ideal teamwork performance even though they had mastered the single-rescuer CPR skill. Poor performance was widespread in task allocation, leadership, and communication. Previous studies have revealed that not only technical skills of individuals but also professional teamwork is required to implement successful resuscitation for patients with OHCA with multiple bystanders on the scene (24). A good resuscitation team should possess skills in leadership, task management, effective communication, mutual supervision, and maintenance of guidelines (25). Consequently, it's necessary to emphasize team-based intervention in the CPR educational plan. Our team-based CPR plan emphasizes mutual support and cooperation throughout the resuscitation, ensuring high-quality CPR through properly allocating and shifting team member roles and providing defibrillation without interfering with basic chest compression and breathing. It has been proved to be effective to improve teamwork performance in this before-after intervention study.

Leadership in a competent team is essential for providing high-quality CPR (26). As multiple bystanders may feel bewildered in such a critical situation, a strong leader is important to calm them down, assign tasks, and coordinate each member during the resuscitation. People who have received education about the team-based CPR plan are strongly recommended to take the responsibility as a team leader, delegating various functions to other untrained bystanders during the resuscitation. To sum up, the educational plan is expected to improve health literacy about public willingness to participate in team-based resuscitation and optimize the effectiveness of multiple bystanders.

The median CCF increased from 58 to 64% after the application of a team-based CPR educational plan. The estimated adjusted linear effect on the odds ratio of survival for a 10% change in CCF was 1.11 (95% confidence interval 1.01 to 1.21) (27). In addition, the guidelines have emphasized that hands-off time during CPR should be as short as possible, and a CCF of at least 60% is associated with better resuscitation outcomes due to the time-sensitivity of OHCA (28). We found the median CCF in the pre-training group didn't reach the recommended standard. This unsatisfactory CCF can be contributed to delayed and intermittent resuscitation performed by multiple bystanders, because of ambiguous duty distribution and ineffective communication. And it is confirmed that effective collaboration of multiple bystanders can improve the CCF by reducing pause time for necessary manipulation, such as identification of OHCA, ventilation, defibrillation, or compression exchange between team members (29). Thus, the cultivation of leadership, communication, and cooperation skills during team-based CPR is highly emphasized in the educational plan to reduce hands-off time as much as possible. Satisfactorily, the team-based CPR educational plan was proven to be effective because there was a significant improvement in CCF after the training of our plan.

Our results revealed that compression depth was continuously deteriorating during 10 min CPR whether trained with the team educational plan or not. As noted, the proportion of adequate compression depth (5−6 cm) significantly improved after Role C took the AED back in the post-training group (Figure 3). Chest compression at an adequate depth has been recognized as an important aspect of high-quality CPR for a better prognosis patients with OHCA (30). It has been proved that compression depth begins to decrease after 90 to 120 s of CPR, and only 18% of the delivered chest compressions were at sufficient depth after 5 min of resuscitation in the previous study (31). The deterioration of compression depth during prolonged CPR can be mainly attributed to the progressive fatigue of bystanders (32). In terms of the substantial improvement following AED retrieval after our plan was applied, it is probably because role C joined in chest compressions instead of just operating AED in most of the pre-training teams, which relieved the fatigue of role A and B.

We portrayed a team-based CPR scenario to follow in a realistic environment. Although the high-fidelity manikin may not represent the diversity of patients' chests and changes in chest resistance during extended CPR (33), the information gained from our educational plan would help the participants perform proper CPR without unnecessary concerns. In the present team-based CPR plan, we educated the knowledge and attitudes toward safety in the resuscitation based on the AHA and ERC guidelines (12, 13). It has been reported that one of the common errors in CPR is failing to compress to an adequate depth (more than 5 cm but no more than 6 cm) (34). According to previous research (35), chest compression depth always decreased along with the time of performance due to fatigue. In the present study, the median depth after the training was 5.3 cm and none of the delivered chest compressions were over 6 cm depth after the 1st minof resuscitation. We encourage the participants to push hard and deep during chest compressions and exchange the role of compression when they are physically tired in the team-based plan (36).

Team-based CPR plans for bystanders in resuscitation education are an important educational strategy with far-reaching application value. Studies about the effect of team-based CPR training on outcomes of patients with OHCA have mainly focused on ACLS so far, but it should extend to include bystanders in public as well (12). Limited health literacy prevents individuals from developing the knowledge, skills, and confidence necessary to engage in emergency preparedness. Although immediate bystander CPR has been shown to increase OHCA survival by two to threefold (37). Only a minority of bystanders are willing to attempt CPR for patients with OHCA because of lack of confidence, fear of harming the victim, and causing legal trouble. This highlights the importance of early bystander intervention and widespread education. Education of closed-loop communication and effective collaboration in resuscitation teams will reduce medical errors by individual rescuers, increase the willingness of trained bystanders to help patients in a life-threatening situation, and give participants the confidence to attempt resuscitation whenever needed (38). So far, a team-based CPR educational plan as an experimental unit has been applied in the WeCan CPR training program which is a thriving public BLS quality and basic CPR training program in China (33). The significant improvement in the teamwork performance and the resuscitation quality has proven the necessity to popularize the team-based CPR educational plan in the community and workplaces.

This study has several limitations. First, there may be selection bias because we recruited medical students aged 18–23 years. We recruited the participants without the clinical practice. Their CPR awareness and skill level were similar to the general students by questionnaire test. The young generation was considered the ideal population to improve health literacy. They are an important target group for CPR education because they are more likely to perform team-based CPR in practice. Second, this study was conducted in resuscitation teams of 3 people. In the real action, there may be more than three laypersons on the scene. One of the critical elements of high-quality CPR for a team is the fluent cooperation among the rescuers to reduce the hands-off time. We suggest a team of more than 3 rescuers could also implement the team-based CPR protocol by shifting the roles of team members. Lastly, the design of the before-after study could reduce the impact of the confounding factors between groups, but we have to admit that the practice effect was present and could result in a motivational bias. For this reason, the skill retention potential of the team-based CPR plan was not evaluated in our present study. Further skill retention investigations are required in the future.

## Conclusions

An innovative team-based CPR educational plan for lay rescuers could significantly improve the CPR quality and reduce the hands-off time of bystanders in terms of teamwork performance in a simulated scenario. This article verified the effectiveness and feasibility of an innovative team-based CPR educational plan for bystanders in the simulation study, which can be widely applied in the future educational program to increase health literacy and improve emergency preparedness for the public in real rescue events.

## Data Availability Statement

The original contributions presented in the study are included in the article/supplementary material, further inquiries can be directed to the corresponding author/s.

## Ethics Statement

The studies involving human participants were reviewed and approved by Joint Research Ethics Board of the Shanghai Jiao Tong University Schools of Public Health and Nursing (SJUPN-201913). The patients/participants provided their written informed consent to participate in this study.

## Author Contributions

LZ contributed to the conception of the study. JX, HY, FQ, and TC contributed significantly to data collection and data analysis. JX, XD, ZG, and ZL performed the drafting of the manuscript. LZ and CW finalized the manuscript. QF provided administrative advice and consultations. All authors contributed to manuscript revision, read, and approved the submitted version.

## Funding

This research was funded by the National Natural Science Foundation of China (Grant No. 72074144), the Public Health Talent Project of the Shanghai Municipal Health Commission (Grant No. GWV-10.2-XD33), and the Innovative Research Team of High-Level Local Universities in Shanghai (Grant No. SHSMU-ZDCX20212801).

## Conflict of Interest

The authors declare that the research was conducted in the absence of any commercial or financial relationships that could be construed as a potential conflict of interest.

## Publisher's Note

All claims expressed in this article are solely those of the authors and do not necessarily represent those of their affiliated organizations, or those of the publisher, the editors and the reviewers. Any product that may be evaluated in this article, or claim that may be made by its manufacturer, is not guaranteed or endorsed by the publisher.

## References

[1] Tan X Wu Q Shao H. Global commitments and China's endeavors to promote health and achieve sustainable development goals. J Health Popul Nutr. (2018) 37:8. 10.1186/s41043-018-0139-z 29650054 PMC5898031

[2] Santana S Brach C Harris L Ochiai E Blakey C Bevington F Kleinman D Pronk N. Updating health literacy for healthy people 2030: defining its importance for a new decade in public health. J Public Health Manag Pract. (2021) 27(Suppl 6):S258–64. 10.1097/PHH.0000000000001324 33729194 PMC8435055

[3] Kitamura T Kiyohara K Sakai T Matsuyama T Hatakeyama T Shimamoto T. Public-access defibrillation and out-of-hospital cardiac arrest in Japan. N Engl J Med. (2016) 375:1649–59. 10.1056/NEJMsa1600011 27783922

[4] Ho AFW De Souza NNA Blewer AL Wah W Shahidah N White AE. Implementation of a national 5-year plan for prehospital emergency care in singapore and impact on out-of-hospital cardiac arrest outcomes from 2011 to 2016. J Am Heart Assoc. (2020) 9:e015368. 10.1161/JAHA.119.015368 33103542 PMC7763405

[5] Chocron R Jobe J Guan S Kim M Shigemura M Fahrenbruch C Rea T. Bystander cardiopulmonary resuscitation quality: potential for improvements in cardiac arrest resuscitation. J Am Heart Assoc. (2021) 10:e017930 10.1161/JAHA.120.017930 33660519 PMC8174211

[6] Takei Y Nishi T Matsubara H Hashimoto M Inaba H. Factors associated with quality of bystander CPR: the presence of multiple rescuers and bystander-initiated CPR without instruction. Resuscitation. (2014) 85:492–8. 10.1016/j.resuscitation.2013.12.019 24384508

[7] Nishi T Maeda T Takase K Kamikura T Tanaka Y Inaba H. Does the number of rescuers affect the survival rate from out-of-hospital cardiac arrests? Two or more rescuers are not always better than one. Resuscitation. (2013) 84:154–61. 10.1016/j.resuscitation.2012.05.026 22705832

[8] Marsch SC Muller C Marquardt K Conrad G Tschan F Hunziker PR. Human factors affect the quality of cardiopulmonary resuscitation in simulated cardiac arrests. Resuscitation. (2004) 60:51–6. 10.1016/j.resuscitation.2003.08.004 14987784

[9] Clarke S Carolina Apesoa-Varano E Barton J. Code Blue: methodology for a qualitative study of teamwork during simulated cardiac arrest. BMJ Open. (2016) 6:e009259. 10.1136/bmjopen-2015-009259 26758258 PMC4716199

[10] McDonald CH Heggie J Jones CM Thorne CJ Hulme J. Rescuer fatigue under the 2010 ERC guidelines, and its effect on cardiopulmonary resuscitation (CPR) performance. Emerg Med J. (2013) 30:623–7. 10.1136/emermed-2012-201610 22851670

[11] Baldi E Contri E Burkart R Borrelli P Ferraro OE Tonani M. Protocol of a multicenter international randomized controlled manikin study on different protocols of cardiopulmonary resuscitation for laypeople (MANI-CPR). BMJ Open. (2018) 8:e019723. 10.1136/bmjopen-2017-019723 29674365 PMC5914707

[12] Greif R Lockey A Breckwoldt J Carmona F Conaghan P Kuzovlev A. European resuscitation council guidelines 2021: education for resuscitation. Resuscitation. (2021) 161:388–407. 10.1016/j.resuscitation.2021.02.016 33773831

[13] Cheng A Magid DJ Auerbach M Bhanji F Bigham BL Blewer AL. Part 6: resuscitation education science: 2020 american heart association guidelines for cardiopulmonary resuscitation and emergency cardiovascular care. Circulation. (2020) 142(16_suppl_2):S551–79. 10.1161/CIR.0000000000000903 33081527

[14] Dong XJ Zhang L Yu YL Shi SX Yang XC Zhang XQ Tian S Myklebust H Li GH Zheng ZJ. The general public's ability to operate automated external defibrillator: a controlled simulation study. World J Emerg Med. (2020) 11:238–45. 10.5847/wjem.j.1920-8642.2020.04.006 33014220 PMC7517391

[15] Clancy CM Tornberg DN. TeamSTEPPS: Assuring optimal teamwork in clinical settings(.). Am J Med Qual. (2019) 34:436–8. 10.1177/1062860619873181 31479300

[16] Freytag J Stroben F Hautz WE Schauber SK Kammer JE. Rating the quality of teamwork-a comparison of novice and expert ratings using the Team Emergency Assessment Measure (TEAM) in simulated emergencies. Scand J Trauma Resusc Emerg Med. (2019) 27:12. 10.1186/s13049-019-0591-9 30736821 PMC6368771

[17] Cooper S Cant R Connell C Sims L Porter JE Symmons M Nestel D Liaw SY. Measuring teamwork performance: validity testing of the team emergency assessment measure (TEAM) with clinical resuscitation teams. Resuscitation. (2016) 101:97–101. 10.1016/j.resuscitation.2016.01.026 26875992

[18] Cooper SJ Cant RP. Measuring non-technical skills of medical emergency teams: an update on the validity and reliability of the team emergency assessment measure (TEAM). Resuscitation. (2014) 85:31–3. 10.1016/j.resuscitation.2013.08.276 24036193

[19] Cooper S Cant R Porter J Sellick K Somers G Kinsman L Nestel D. Rating medical emergency teamwork performance: development of the team emergency assessment measure (TEAM). Resuscitation. (2010) 81:446–52. 10.1016/j.resuscitation.2009.11.027 20117874

[20] Sedgwick P. Multiple hypothesis testing and Bonferroni's correction. BMJ. (2014) 349:g6284. 10.1136/bmj.g6284 25331533

[21] Chien CY Fang SY Tsai LH Tsai SL Chen CB Seak CJ. Traditional versus blended CPR training program: a randomized controlled non-inferiority study. Sci Rep. (2020) 10:10032. 10.1038/s41598-020-67193-1 32572100 PMC7308401

[22] Kim S Ahn KO Jeong S. The effect of team-based CPR on outcomes in out of hospital cardiac arrest patients: a meta-analysis. Am J Emerg Med. (2018) 36:248–52. 10.1016/j.ajem.2017.07.089 28793963

[23] Park JH Moon S Cho H Ahn E Kim TK Bobrow BJ. Effect of team-based cardiopulmonary resuscitation training for emergency medical service providers on pre-hospital return of spontaneous circulation in out-of-hospital cardiac arrest patients. Resuscitation. (2019) 144:60–6. 10.1016/j.resuscitation.2019.09.014 31550494

[24] Andersen PO Jensen MK Lippert A Østergaard D. Identifying non-technical skills and barriers for improvement of teamwork in cardiac arrest teams. Resuscitation. (2010) 81:695–702. 10.1016/j.resuscitation.2010.01.024 20304547

[25] Pearson DA Darrell Nelson R Monk L Tyson C Jollis JG Granger CB Corbett C Garvey L Runyon MS. Comparison of team-focused CPR vs standard CPR in resuscitation from out-of-hospital cardiac arrest: Results from a statewide quality improvement initiative. Resuscitation. (2016) 105:165–72. 10.1016/j.resuscitation.2016.04.008 27131844

[26] Yeung JH Ong GJ Davies RP Gao F Perkins GD. Factors affecting team leadership skills and their relationship with quality of cardiopulmonary resuscitation. Crit Care Med. (2012) 40:2617–21. 10.1097/CCM.0b013e3182591fda 22732290

[27] Christenson J Andrusiek D Everson-Stewart S Kudenchuk P Hostler D Powell J. Chest compression fraction determines survival in patients with out-of-hospital ventricular fibrillation. Circulation. (2009) 120:1241–7. 10.1161/CIRCULATIONAHA.109.852202 19752324 PMC2795631

[28] Panchal AR Bartos JA Cabanas JG Donnino MW Drennan IR Hirsch KG. Part 3: adult basic and advanced life support: 2020 american heart association guidelines for cardiopulmonary resuscitation and emergency cardiovascular care. Circulation. (2020) 142(16_suppl_2):S366–468. 10.1161/CIR.0000000000000916 33081529

[29] Jiang C Zhao Y Chen Z Chen S Yang X. Improving cardiopulmonary resuscitation in the emergency department by real-time video recording and regular feedback learning. Resuscitation. (2010) 81:1664–9. 10.1016/j.resuscitation.2010.06.023 20727657

[30] Stiell IG Brown SP Christenson J Cheskes S Nichol G Powell J. What is the role of chest compression depth during out-of-hospital cardiac arrest resuscitation? Crit Care Med. (2012) 40:1192–8. 10.1097/CCM.0b013e31823bc8bb 22202708 PMC3307954

[31] Sugerman NT Edelson DP Leary M Weidman EK Herzberg DL Vanden Hoek TL. Rescuer fatigue during actual in-hospital cardiopulmonary resuscitation with audiovisual feedback: a prospective multicenter study. Resuscitation. (2009) 80:981–4. 10.1016/j.resuscitation.2009.06.002 19581036 PMC2746377

[32] Dong X Zhou Q Lu Q Sheng H Zhang L Zheng ZJ. Different resting methods in improving laypersons hands-only cardiopulmonary resuscitation quality and reducing fatigue: a randomized crossover study. Resusc Plus. (2021) 8:100177. 10.1016/j.resplu.2021.100177 34825237 PMC8605240

[33] Dong X Zhang L Myklebust H Birkenes TS Zheng ZJ. Effect of a real-time feedback smartphone application (TCPRLink) on the quality of telephone-assisted CPR performed by trained laypeople in China: a manikin-based randomised controlled study. BMJ Open. (2020) 10:e038813 10.1136/bmjopen-2020-038813 33023877 PMC7539615

[34] Soar J Perkins GD Nolan JP. Chest compression quality–push hard, push fast, but how deep and how fast? Critical Care Med. (2012) 40:1363–4. 10.1097/CCM.0b013e31824102e2 22425844

[35] Liu S Vaillancourt C Kasaboski A Taljaard M. Bystander fatigue and CPR quality by older bystanders: a randomized crossover trial comparing continuous chest compressions and 30:2 compressions to ventilations. CJEM. (2016) 18:461–8. 10.1017/cem.2016.373 27650514

[36] Travers AH Perkins GD Berg RA Castren M Considine J Escalante R. Part 3: Adult basic life support and automated external defibrillation: 2015 international consensus on cardiopulmonary resuscitation and emergency cardiovascular care science with treatment recommendations. Circulation. (2015) 132(16 Suppl 1):S51–83. 10.1161/CIR.0000000000000272 26472859

[37] Iwami T Kawamura T Hiraide A Berg RA Hayashi Y Nishiuchi T. Effectiveness of bystander-initiated cardiac-only resuscitation for patients with out-of-hospital cardiac arrest. Circulation. (2007) 116:2900–7. 10.1161/CIRCULATIONAHA.107.723411 18071072

[38] Rosen MA DiazGranados D Dietz AS Benishek LE Thompson D Pronovost PJ Weaver SJ. Teamwork in healthcare: key discoveries enabling safer, high-quality care. Am Psychol. (2018) 73:433–50. 10.1037/amp0000298 29792459 PMC6361117

